# Universal food security program and nutritional intake: Evidence from the hunger prone KBK districts in Odisha

**DOI:** 10.1016/j.foodpol.2016.07.003

**Published:** 2016-08

**Authors:** Andaleeb Rahman

**Affiliations:** Indian Institute for Human Settlements (IIHS), Bangalore, India

**Keywords:** Consumer subsidy, Nutrition, Program evaluation, Hunger, India

## Abstract

This article provides evidence on the role of consumer food subsidies in improving nutritional intake and diet quality by evaluating the expansion of the government food assistance program coverage in the hunger prone state of Odisha in India. In 8 districts of Odisha, popularly known as the Kalahandi-Balangir-Koraput (KBK) region which is notable for extreme poverty and starvation deaths, the government did away with the targeted food assistance program in 2008 and made the scheme universal. Using a Difference-in-Difference methodology over two repeated cross sectional household surveys, this article finds that the shift from targeted to a universal food security program in the KBK region of Odisha has led to an improvement in the household nutritional intake and diet quality. Further examination suggests that proportion of households consuming below the recommended dietary allowance of calorie, fats and protein has declined significantly in this region post the intervention.

## Highlights

• This paper provides evidence on the link between consumer subsidy and nutrient intake.
• We compare districts with a targeted versus universal subsidy entitlements.
• Universalization of PDS in some of the poorest districts led to greater nutrient intake and diet quality.
• These results have important implications for the National Food Security Act in India.

## 1 Introduction

In order to address the problem of undernutrition, particularly among the poor, consumer food subsidies are an important policy instrument in many of the low income countries. Social protection measures such as the food assistance programs have a crucial role to play in promoting greater nutrient intake and hence overall nutrition (Lentz and Barrett, 2013). Such measures have become important since growth in income, an essential driver of improved nutritional outcomes, has not translated into a proportionate decline in hunger and malnourishment (FAO, WFP, and IFAD, 2013). In such an environment, the provision of staple food at subsidized prices not only increases access to food to the beneficiaries but also provides them an implicit income transfer which is the difference between the open market and subsidized price for every unit of the food item purchased. Whether this gain in income would translate into consumption of a nutritious basket of food items has been a much debated issue with limited empirical evidence. Theoretically, price subsidies would have a positive effect if the income gain is spent on the consumption of more nutritious items. On the other hand, if households substitute away from less costly staple food items towards those which are tastier but less nutritionally less dense, consumer subsidies would lead to a decline in the nutrient intake. Using data from a randomized field experiment in China, Jensen and Miller (2011), found that households which are provided the food subsidy substitute away from the staple food towards those food items which are expensive but low on nutrient content leading to a reduction in the overall calorie intake. In the case of China, Shimokawa (2010) finds that consumer subsidies have an asymmetric effect on nutrient intake. While an increase in consumer food subsidies positively affects the total energy intake, removal of the subsidies leaves the energy intake unaffected.

In India, the central government provides subsidized food grains to the poorer households under the Public Distribution System (PDS) which is amongst the largest food security programs in the world. The effectiveness of the PDS has been debated heavily on account of two reasons—its failure to reach the poor and the escalating costs of operation. However, in recent years, there has also been an improvement in the functioning of PDS with the state governments taking various measures to make delivery more effective and enhance the PDS coverage. Dreze and Sen (2013) refer to this as the “new style” PDS in which the state governments provide grains at extremely low prices to a larger section of the population. While PDS has historically worked well in Tamil Nadu and Himachal Pradesh, the turnaround in the functioning of the PDS is evident even in the poorer states of Chhattisgarh and Odisha.

Given the revival of PDS, an immediate question of interest is whether the subsidies through PDS impacts overall nutrient intake and diet quality. As we will discuss in Section 2, the last word is far from being written on this issue. One of the early studies evaluating the effectiveness of PDS was by Kochar (2005). Using cross-sectional data in two time points − 1993–94 and 1999–00 during which PDS became a scheme targeted towards a poorer from a universal scheme, Kochar investigates whether consumption from PDS had an effect on total calorie consumed by the poorer households. She finds that greater income transfers through the PDS did not lead to greater calorie consumption for the targeted households. Krishnamurthy et al. (2014a) and Kishore and Chakrabarti (2015) study the recent improvements in the PDS delivery and show how the expansion of its coverage to a larger share of the population led to improvements in diet quality.

In this paper, we explore the link between access to PDS and nutrient consumption in the state of Odisha.^1^ In 8 districts of the state, PDS was declared universal while it remained a targeted scheme in the other 22 districts of Odisha. We use repeated cross-section data and exploit this unique natural experiment to estimate the impact of PDS on nutrient intake and diet quality. While doing so, we make the following contributions. First, we provide evidence on the role of publicly provided assistance program in improving nutrient intake and diet quality in a state with low level of economic development and regional disparity.^2^ Second, we contribute to the debate on targeted versus universal food security scheme by comparing two regions with either of these schemes within the same state. Third, when analyzing the impact of PDS on the intake of nutrients, the focus of our analysis goes beyond the total energy intake as measured through the consumption of calories. In addition to calories, we also focus on other major macro-nutrients—protein and fat. Using the recommended intake of these macronutrients, as determined by the Indian Council for Medical Research (ICMR), we also look at whether households are moving closer towards the Recommended Dietary Allowance (RDA) of nutrients as a result of consumption from PDS. To get a better sense of dietary quality, we break down the consumption of calories into its various sources: cereals, pulses, fruits and vegetables, eggs, fish and meat, edible oil and others.

**Fig. 1:**
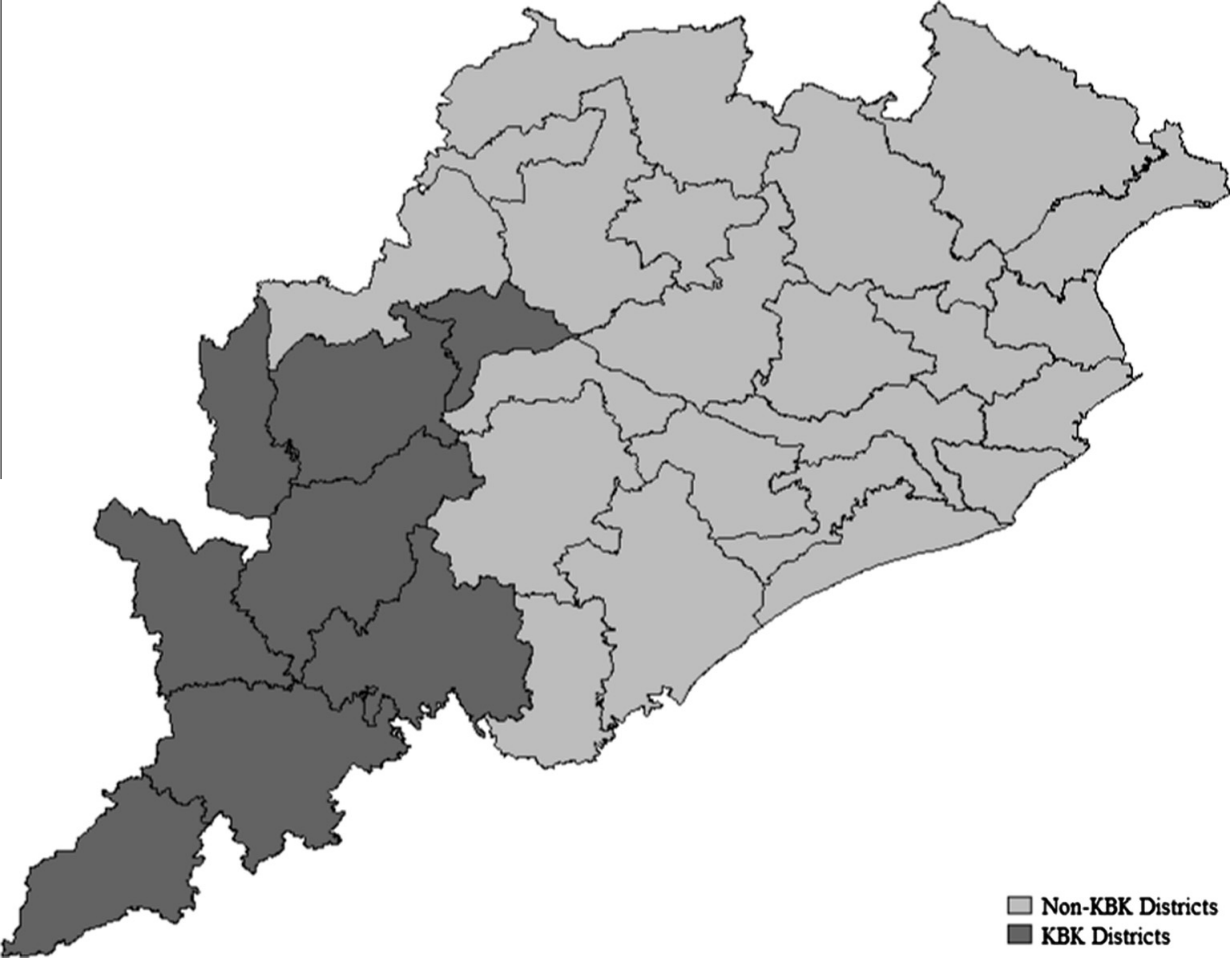
Odisha and KBK districts.

**Fig. 2:**
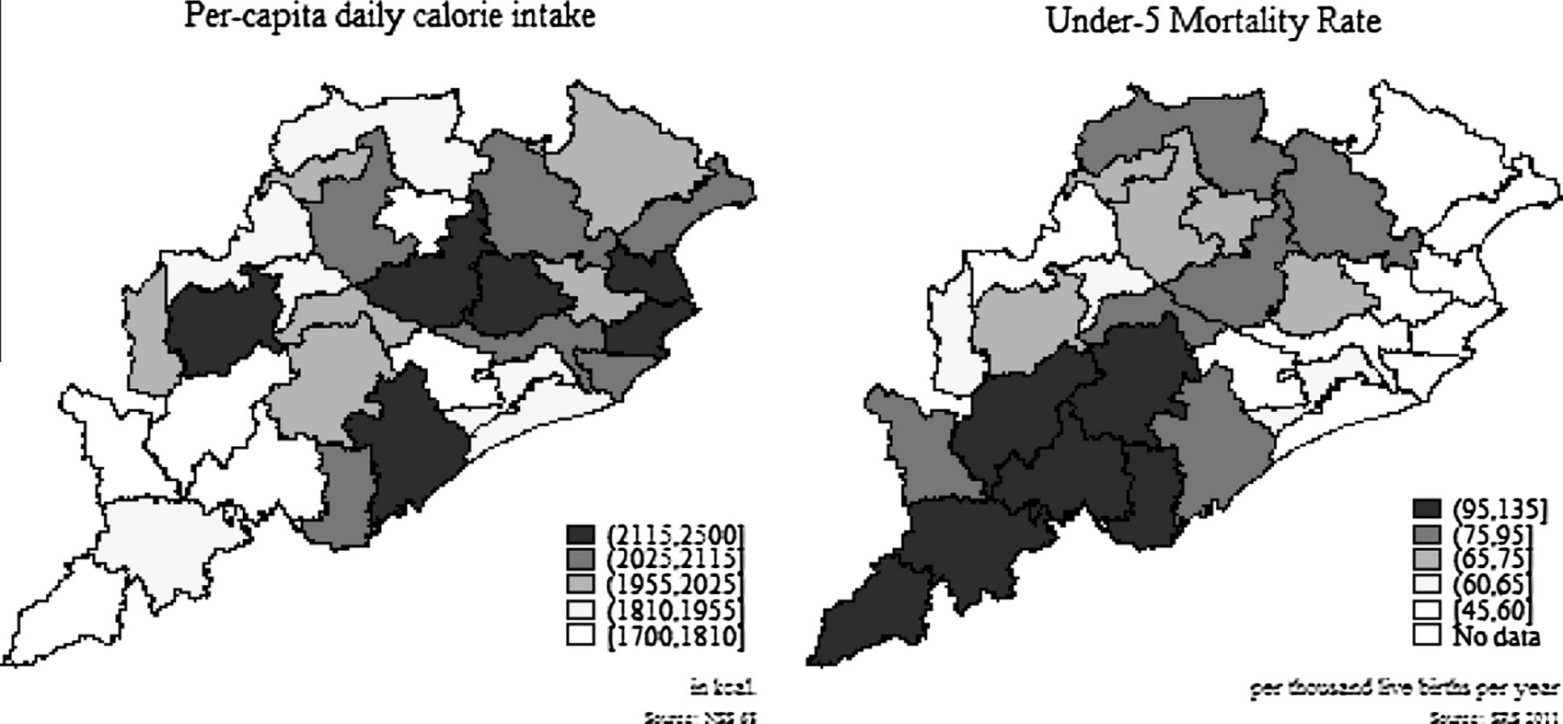
Per-capita calorie intake and infant mortality rates for Odisha.

In this paper, we focus on Odisha since it is one of the poorest states in India and suffers from “alarming” level of hunger (Menon et al., 2009). In 2011–12, 17.29 percent of the urban and 35.69 percent of the rural population were found to be poor as per the official poverty line. High level of food insecurity is evident in the form of higher mortality and under-nutrition, especially amongst the scheduled tribes (STs) and the scheduled castes (SCs). There is also considerable disparity within the state- across social groups and regions.^3^ The KBK region which comprises of 8 contiguous districts in the southern part of Odisha (Fig. 1) is amongst the poorest regions in the country owing to their vulnerability to droughts and floods (Kujur, 2006, Parida, 2008, Shah et al., 2008).^4^ These districts are also characterized by poor nutrient intake and higher infant mortality ratio (Fig. 2). Recognizing the poor nutritional indicators and higher poverty levels in the KBK region, the government of Odisha decided to move away from a PDS targeted towards the poor to a universal one in the KBK region in 2008. Since all the households living in the KBK districts are now eligible for the subsidized rice through PDS, this led to an increase the number of beneficiaries from approximately 30 lakh to 55 lakh (Wadhwa, 2009). Extra allocation for the increase in the number of beneficiaries was made by reducing the PDS entitlements from 35 kg to 25 kg of subsidized rice for the identified poorer households. Given that in the non-KBK region of the state, PDS is not universal, there are differential levels of implicit income transfer across these two regions of Odisha. In the KBK region, income transfers are higher than the non-KBK districts. It is this variation in implicit income transfer over time that we exploit to evaluate the link between consumer subsidies through PDS and nutrient intake.

This paper is organized in the following way. Section 2 discusses the existing evidences on PDS and nutrient intake followed by a description of the data and the summary statistics in Section 3. Econometric methodology and the identification strategy is discussed in Section 4. Results are presented in Section 5 and following section concludes the analysis.

## 2 Existing evidences on the link between PDS and nutrition

The link between consumer food subsidy and nutrient intake is theoretically ambiguous (Jensen and Miller, 2011). The impact of consumer subsidy overall nutrient intake depends upon how consumers choose to substitute among the various food items. Consumer subsidies lead to an increase in the implicit income transfer. If the households choose to spend it on more nutritious items, it may lead to an increase in total nutrient intake. At the same time, consumers may use this income gain to consume more of other food products which may not be nutritious enough. Hence, consumer food subsidies may also lead to substitution effects if the consumers choose to consume more expensive food items which are nutritionally less dense. This may be on account of their preference for variety in the diet over more nutritious staple food items. This leads to an unresolved empirical question of whether the income or the substitution effect dominates when it comes to increase in implicit income transfer as a result of consumer food subsidies.

Much of the literature on the nutritional impact of consumer food subsidies has focused on the link between PDS and per capita consumption of calories since subsidized foodgrains is expected to improve the total energy intake of households. Existing literature on the link between PDS and nutrient intake basically looks at the two major events of reform in the history of PDS − the decision to make PDS a targeted scheme only towards the poorer households from a universal one in 1997; and the recent initiatives by the various state government to improving its functioning. Kochar (2005) examined the outcome of greater consumer subsidy or implicit income transfer to the poorer households owing to the change in PDS from a universal to a targeted scheme in 1997. Using cross-sectional household data for the years 1993–94 and 1999–00, she finds that the greater wheat subsidy to the poorer households did not lead to an improvement in their overall calorie intake. Kaushal and Muchomba (2015) also evaluate the impact of the transition from universal to a targeted PDS on the nutritional intake using nationally representative data for the period 1993–94 and 2009–10. While the sample of states in Kochar (2005) was restricted only to the wheat consuming states, Kaushal and Muchomba (2015) expand the sample by including the rice consuming states as well, since rice consuming states have traditionally had a better functioning PDS compared to rice consuming ones. Both these studies find a negligible effect of consumer subsidy from PDS on total calorie intake. They find that though the consumption of calories from rice and wheat increased, but the consumption of more nutritious coarse cereals declined.

Contrary to finding of these earlier studies, evaluating the PDS reforms in the state of Chhattisgarh between 1999–00 and 2004–05, Krishnamurthy et al., 2014a, Krishnamurthy et al., 2014b find that greater coverage of PDS has not only increased the intake of calories, but improved the quality of diet as well. Household now consume a greater share of their calories from pulses and other animal-based proteins. Kishore and Chakrabarti (2015) compare five states of India which have expanded the coverage of households under PDS between 2004–05 and 2009–10, with the rest of the states. They find that the expansion of PDS coverage in these states led to greater consumption per household from the PDS. The greater implicit income transfer as a result is used to spend on other nutritious food items − pulses, edible oils and vegetables, suggesting an improvement in the dietary quality. Kaul (2013) compares the impact of implicit income transfer due to PDS and an equivalent increase in the household expenditure on calorie intake of households. She finds that 1 percent increase in the transfer due to PDS would increase caloric intake by 0.14 percent, suggesting a positive effect.

The estimation methodology as adopted in Kochar (2005) and Kaushal and Muchomba (2015) have their limitations. Kochar (2005) compares the change in nutrient intake of the poorer households after PDS became a targeted scheme. But, the baseline survey which she uses does not have information on whether the household was officially classified as poor or not. Based upon certain observable characteristics of the household, Kochar (2005) estimates the probability of a household being poor. Jensen and Miller (2011) argue that such an identification of the poor households is incorrect. Incorrect identification of poor and non-poor households may bias the result towards finding a statistically insignificant relationship between nutrient intake and consumer food subsidy. Kaushal and Muchomba (2015) encounter a similar problem. In the absence of any information that identifies a household as poor in the survey data, they also use a regression method to estimate the predicted probability of a household being poor. Recognizing the impreciseness of their identification method, they restrict their sample to only those households with monthly per capita expenditure (MPCE) less than the median to ensure a reasonable comparison. The limitations of Kochar’s (2005) study, viz. the calculation of the probability of being a poor household, is also valid here. In the absence of any information on the household being poor, Kishore and Chakrabarti (2015) assume that the households in the bottom 20 percent the MPCE decile to be poor. The identification strategies employed in these papers to establish a causal impact of PDS on nutrient intake are based on stringent assumptions.

Here, in this paper, we have estimated and shown the impact of PDS on nutrient intake indicators with much less restrictive assumptions since we can identify the households as poor and non-poor based upon the information on the possession of ration cards in both time periods.

## 3 Data and descriptive statistics

Data used in this paper comes from two rounds of nationally representative consumer expenditure surveys as carried out by the National Sample Survey Organization (NSSO) in 2004–05 and 2011–12. The survey has household level information on the quantity consumed of a range of food and non-food items and the expenditure incurred on them in the last 30 days preceding the survey.^5^ Quantity and expenditure information on the items consumed from PDS like rice, wheat, sugar and kerosene are also collected as a part of the surveys. These surveys also contain information on the monthly per-capita expenditure (MPCE) and other socio-economic characteristics of the households including their geographical location, social group, religion, demographic composition of the household, type of ration card held and the durable goods possessed. These surveys are representative at the district level for rural as well as urban areas (Chaudhuri and Gupta, 2009).

We analyse two rounds of the data with information on 3819 and 2973 rural households in Odisha for the years 2004–05 and 2011–12 respectively. The 2004–05 survey acts as a baseline since a universal PDS in Odisha came into being in 2008 while the information from 2011–12 survey captures the post-intervention outcomes. The impact of PDS is quantified here using the cross-sectional variation over time. The sample is restricted to rural areas of Odisha since the PDS revival has been more effective in the rural areas.

As per standard practice, we convert the consumption of food items into their nutrient content (calorie, protein and fat) using the nutrient value of Indian food item.^6^ according to Gopalan et al. (1971) To examine the source of nutrients and the variety of food items in the diet, food consumption is sub-divided into the following six groups: cereals, pulses, dairy products, eggs, fish and meat, fruits and vegetables, edible oils and other food items. ICMR RDAs are at an individual level, and vary by age, gender, weight and nature of work of the individual. Since, our analysis is at the household level, we have converted the RDA norms into aggregate household energy requirements based upon the household demographic profile as identified in our dataset.^7^

### 3.1 Descriptive statistics

Poverty levels in Odisha are much higher than the rest of the country. According to the 2011–12 Planning Commission estimate, 35.7 percent of the rural population in Odisha can be classified as poor, which is 10 percentage points higher than the all India estimates of 25.7 for rural poverty. There is wide disparity within Odisha as well. We estimate that the MPCE stood at Rs. 294.95 in the KBK districts as compared to Rs. 415.32 in the rest of Odisha at 2004–05 constant prices. The KBK districts continue to have a lower level of expenditure in 2011–12 as well but the gap between MPCE of the KBK and non-KBK districts has narrowed down between 2004–05 and 2011–12.

To be able to access the PDS, households need to possess a ration card. Ration card is a document issued by the government which entitles an individual/family to purchase from the PDS. Ration cards are also used as an identity card to avail many of the other government schemes, since it classifies households based upon their poverty status. Ration cards are of three types- Antayodaya Yojana Yojana (AAY) card for the poorest of the poor, Below Poverty Line (BPL) card for the poor and Above Poverty Line (APL) card for those households who are not identified as poor. Looking at the distribution of ration card across the state, we find that 33 percent of the households in 2004–05 did not have a ration card and this declined to 28 percent in 2011–12 (Table 1).^8^ It is to be noted that in spite of a reduction of the number of the households with no ration card in the districts belonging to the KBK region, a sizable share of the households (27.07 percent) are outside the ambit of PDS with no ration card. Share of households with AAY and BPL cards in Odisha has gone up over time while the share of households with the APL cards has come down. This is more pronounced in the KBK districts where there has been a 10 percentage point increase in the coverage of BPL cards between 2004–05 and 2011–12, compared to 5 percentage point in the rest of Odisha. Similarly, in the KBK districts, we have seen an increase in the percentage of households with APL cards, while this has declined in the non-KBK districts.

In line with the improvement in coverage under PDS as reflected by issuance of ration card, there has been a substantial increase in the quantity as well as share of rice consumed from PDS.^9^ In the KBK districts, average household consumption of rice from PDS has increased from 8.9 kg to 20 kg per month (Table 2). In the non-KBK districts, there has been an almost five-fold increase from 3.3 kg to 15.1 kg In terms of the share, monthly consumption of rice from PDS to the total rice consumption increased from 19 percent to 44.5 percent in KBK districts. We find that the increase in consumption of rice from PDS for the AAY households is lower compared to the BPL households since the emphasis on targeting the poorest of the poorest households has always been there. Similarly, BPL households in the KBK districts consumed a larger amount of rice from PDS as compared to other districts and hence the scope for improvements in the delivery was higher in the latter. Still, we see observe a doubling of the consumption of rice from PDS in the KBK districts for the BPL households. With the removal of any distinction between the APL and BPL card holders in the KBK district, the average consumption of rice from PDS for the APL households has gone up from 6.4 kg in 2004–05 to 21.9 kg in 2011–12. Similarly, the share of rice from PDS increased from 11.6 to 40.5 percent.

**Fig. 3:**
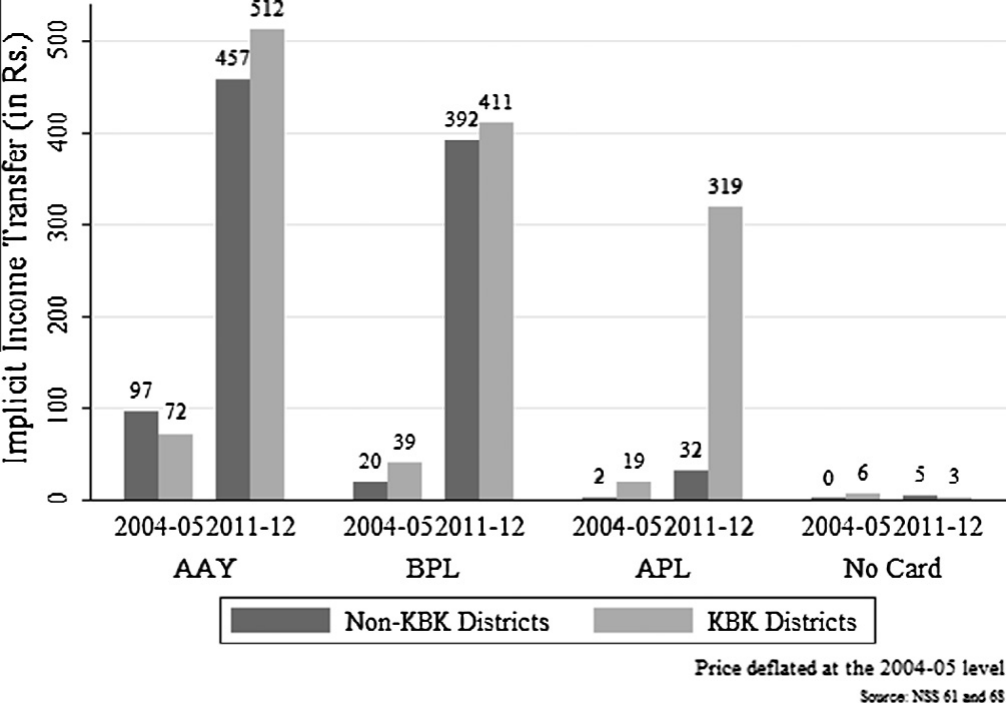
Average monthly implicit income transfer to the households.

In line with the literature we calculate the implicit income transfer as the product of the difference between the market price, *p_m_* as proxied by the unit value and PDS price *p_d_* and the quantity *q_d_* consumed from the PDS, which is (*p_m_* − *p_d_*) ∗ *q_d_*.^10^ We can see a clear increase in the implicit income transfer for both the AAY and BPL card holders over time (Fig. 3). For the AAY and BPL households in the KBK districts, there has been an increase in the monthly income transfer of Rs. 440 and Rs. 372 respectively between 2004–05 and 2011–12. In the non-KBK districts, implicit income transfer increased by Rs. 360 and Rs. 372 for the AAY and BPL households respectively. For the APL households, there is perceptibly little income transfer in the non-KBK districts in both the time periods, but there is a substantial increase for the APL household in the KBK region. Monthly implicit income transfers to APL households in the KBK districts increased from Rs. 32 per household in 2004–05 to Rs. 319 in 2011–12. This reflects the fact that greater PDS entitlements to the APL households in the KBK districts had a clear impact on their consumption of rice from PDS.

Average per-capita consumption of calorie, protein and fat is lower in the KBK region of Odisha as compared to the non-KBK districts (Table 3). In 2011–12, mean per-capita daily calorie intake was 1819 kcal in districts belonging to the KBK region as compared to 2046.5 kilocalories (kcal) in non-KBK region. Similar pattern exists for fats and protein. For households differentiated on the basis of ration cards, those with the APL card are better off than others in terms of nutrient intake. AAY card holders in the KBK region consumed a lower amount of fat than the BPL households, but their daily intake of calorie and protein is higher. There is no apparent difference in the consumption of calorie, protein and fat between the AAY and BPL households in the non-KBK districts.

In Table 4, we compare changes in the average nutrient intake over the two survey rounds using the *t-test*. Since, the AAY households constitute a small proportion of our sample and they are also poor, we include them in the BPL category here. In addition to the major macronutrients- calorie, protein and fat, we report the consumption of calories from the major food groups as described earlier. Overall, there is an increase in the intake of calories. But, we do not find to be statistically significant. For the BPL households, this increase is not only larger but statistically significant as well (Table 4). Increase in consumption of calories is larger for the BPL households belonging to the KBK region (237.8 kcal as compared to 45.1 kcal in the non-KBK region). In the KBK region, though there is an overall increase in the calorie intake for the BPL as well as the non-BPL households, it is not statistically significant for the latter. In the non-KBK region, calorie intake has increased only for the BPL households while there is a decline for the non-poor households.

Over the period 2004–05 and 2011–12, change in the consumption of protein is broadly similar to that of calories, but the intake of fat has increased for all household categories irrespective of which region of Odisha they belong to. Increase in the consumption of fat is higher for the BPL households across the regions. One possible reason could be their low level of fat intake earlier. Sources of calorie is an important barometer to measure diet quality. Since cereals are the staple diet in the region, a shift away from them towards more varied items would signal an improvement in diet. The intake of calorie from cereals has declined over the period for both the poor and non-poor households in the non-KBK districts but the opposite holds true for the KBK region. In terms of calorie from non-cereals, there is an across the board increase. Pulses as a source of calories has increased in importance together with the dairy products and edible oils for the households (Table 4).

There is perceptibly little change in the proportion of households with calorie consumption below their RDA norms in the KBK districts over time on an average (Table 5). But, across MPCE quartiles, we can see that for households in lower quartiles, a lower proportion of population is consuming below their RDA which suggests an overall increase in the calorie intake for them. In the case of non-KBK districts, we find an opposite trend with the proportion of households consuming less than their RDA of calories across all quartiles. For the case of protein, we find a decline in the percentage of households consuming below their RDA across both the KBK and non-KBK districts. In terms of fat intake, all households in the first and second MPCE quartiles of the KBK districts are found to be consuming below their RDA. For the higher quartiles, we find lesser proportion of population consuming less than their recommended fat intake. Increase in the calorie and protein consumption over time in the KBK district contrasts with the all-India trend which shows a secular decline in the consumption of calories and protein over time.^11^ This underscores the fact that households in the KBK districts are much poorer and consume lesser nutrient compared to the rest of the country.

## 4 Empirical strategy

Difference-in-Difference (DID) approach has been a standard method in the literature to investigate the impact of PDS on nutrient intake (Kishore and Chakrabarti, 2015, Krishnamurthy et al., 2014a, Krishnamurthy et al., 2014b). It is a useful tool to establish causal effect of an intervention when there is a baseline and follow-up information in the form of repeated cross-section (Khandker et al., 2009, Imbens and Wooldridge, 2009). In a DID set-up, mean outcome of the treatment group before and after the intervention is compared. The difference between observed changes in the mean outcome of the treatment and control group after the intervention is the DID estimate. Assume $Y_{0}^{T}$ and $Y_{1}^{T}$ represent the mean outcome of the treatment group before and after the intervention respectively. Similarly, let, $Y_{0}^{C}$ and $Y_{1}^{C}$ be the respective mean outcome of the control group post and prior to the intervention. Then, the DID estimate is given by:

(1) $$\tau_{\mathrm{DID}}=E(Y_{1}^{T}-Y_{0}^{T}|T=1)-E(Y_{1}^{C}-Y_{0}^{C}|T=0)$$

Here *T* = 1 implies the treatment while *T* = 0 stands for no treatment.

In the parlance of evaluation methods, KBK region—with a universal PDS—is our treatment group while the rest of Odisha is the control group. The 2004–05 survey is our baseline while the 2011–12 survey one is the post-intervention information for the given treatment and control groups. Non-KBK districts are the closest one would get to a treatment group for the KBK regions. Krishnamurthy et al., 2014a, Krishnamurthy et al., 2014b while evaluating the PDS reforms in the state of Chhattisgarh use the neighboring districts as the control group while the whole of the Chhattisgarh acts as the treatment group. Here, we are using the non-KBK districts within the same states as our control group since the degree of improvement, governance and entitlements differ widely across states. For further robustness checks, we restrict our sample only to the KBK districts and consider households without any ration card as the alternative control group, with all other households with a ration card (AAY/BPL/APL) as the treatment group. This is justified since the households without any ration card have per-capita expenditure and other characteristics similar to the other households in this region. Also, the percentage of households without a ration card constitutes a substantial proportion of the population as shown in Table 1.

The DID approach has its distinct advantages over the other methods of causal identification especially in the case of repeated cross-sectional datasets when the selection takes place on unobservable factors. Double differencing as shown in (1) removes that bias in the post-intervention comparison between the treatment and control groups which may be due to any permanent differences between, also called as the time-invariant factors (Imbens and Wooldridge, 2009). But care must be taken to control for the unobserved factors which affect participation and vary over time. The foremost assumption while considering *τ_DID_* to be true causal effect of an intervention is that of unconfoundedness. It basically implies that conditional on a set of observable factors, there are no unobserved factors which could affect the potential outcomes (Imbens and Wooldridge, 2009). In a non-random setting, this assumption is unlikely to hold since selection or participation in any program is hardly based upon all the factors which are observable to the researcher. This induces selection bias into program evaluation. For causal identification, we need to control for both the time-varying and time-invariant unobservable factors which might affect the outcome variable. The present case is of purposive program placement by the government as the decision of make PDS a universal program in the KBK region was based upon its history of poor nutritional outcome. Hence, the selection of districts into the program (here, PDS) is not random. We do a slew of robustness check to ensure that we control for this later in the paper.

The DID estimate can be captured in a regression framework using the following specification:

(2) $$Y_{\mathrm{idt}}=\beta T_{d}+\tau_{\mathrm{DID}}(T_{d}*t_{i})+\gamma t_{i}+\lambda X_{\mathrm{idt}}+\mu_{d}+\varepsilon_{\mathrm{idt}}$$

*Y_idt_* is the observed outcome variable for household *i* in district *d* at time *t*. *T_d_* is the dummy for treatment region and *t_i_* is the time dummy. The coefficient *τ_DID_* on the interaction term between time and treatment dummy is the DID estimate. The other household level factors *X_idt_* can also be controlled for in the regression in addition to the district fixed effects, *μ_d_*. To control for the time in-variant heterogeneity, we use the district fixed effects in the regressions. Controlling for the time-variant heterogeneity, which is the unobserved factors affecting program participation over time is quite challenging in a repeated cross-section. It basically implies that the outcomes in the control as well as treatment groups would have followed the same trend in the absence of an intervention, even though the mean outcome may be different. The commonly used, but a coarse method to test for this has been to check for the parallel trend assumption. In statistical terms, parallel trends assumption holds if the DID estimate, *τ_DID_* is statistically insignificant when we run the same regression with data from the baseline and an earlier period (pre baseline).

While we test for the link between expanded PDS coverage to non-poor households, and its impact on nutrition, another key question of interest which we investigate here is how this has impacted the nutrient intake of the poor households in the KBK region. It has been argued that a universal PDS in place of a targeted one would increase welfare of the poor as well since broader coverage would reduce the exclusion errors of targeting (Himanshu and Sen, 2011, Kotwal, 2011). This line of argument broadly follows from the political economy literature which says that the effectiveness of any public program depends upon the amount of benefit it bestows upon the non-poor. The better off sections of the population have a greater political support and voice and hence any public program targeted specifically at the poor runs the risk of reduced political support (Kanbur and Besley, 1990, Gelbach and Pritchett, 2001, Gelbach and Pritchett, 2002). To ascertain whether the expansion of PDS coverage to the non-poor households impacted the poorer households in KBK districts vis a vis the non-KBK districts, a triple DID regression is employed wherein the time dummy, *t_i_* is interacted with the treatment dummy (*T_d_*) for the KBK region here and a dummy for the households with a BPL card (*BPL_i_*). Triple DID estimation uses a regression approach as represented in Eq. (3). We are interested in the triple DID estimator, $\tau_{\mathrm{DID}}^{\mathrm{tr}}$ which gives a measure of the move towards universal PDS in KBK districts on its BPL population. As represented in Eq. (4), by subtracting the change over time for the BPL households in the non-KBK region, i.e. $E(Y_{1\text{,}\mathrm{BPL}}^{C}-Y_{0\text{,}\mathrm{BPL}}^{C}|T=0)$ and other non-BPL households in KBK districts viz. $E(Y_{1\text{,}\mathrm{oth}}^{T}-Y_{0\text{,}\mathrm{oth}}^{T}|T=1)$ from changes in BPL households belonging to the KBK districts, $\tau_{\mathrm{DID}}^{\mathrm{tr}}$ informs us of the true impact of the removal of APL-BPL difference in the KBK region upon the BPL households.

(3) $$Y_{\mathrm{idt}}=\beta T_{d}+\tau_{\mathrm{DID}}^{\mathrm{tr}}(T_{d}*t_{i}*{\mathrm{BPL}}_{i})+\gamma t_{i}+\delta (T_{d}*{\mathrm{BPL}}_{i})+\theta (t_{i}*{\mathrm{BPL}}_{i})+\lambda X_{\mathrm{idt}}+\mu_{d}+\varepsilon_{\mathrm{idt}}$$

(4) $$\tau_{\mathrm{DID}}^{\mathrm{tr}}=E(Y_{1\text{,}\mathrm{BPL}}^{T}-Y_{0\text{,}\mathrm{BPL}}^{T}|T=1)-E(Y_{1\text{,}\mathrm{BPL}}^{C}-Y_{0\text{,}\mathrm{BPL}}^{C}|T=0)-E(Y_{1\text{,}\mathrm{oth}}^{T}-Y_{0\text{,}\mathrm{oth}}^{T}|T=1)$$

The outcome variable in the above econometric specification are the major macronutrients- calorie, fat and protein in daily per-capita terms. In addition to that, we look at the amount of calorie consumed through different food source- pulses, dairy products, eggs, fish and meat, vegetable and fruits, edible oils and others. In the regressions, we use a logarithmic transformation for the nutrient intake. We construct the ratio of actual nutrient intake to the RDA of calorie, fat and protein per adult equivalent and see whether PDS has played a role in ensuring that households are now closer to the RDA. It is essential to control for the socio-economic and other demographic characteristics since the nutritional status of the households are not invariant to them. Chronic energy deficiency is found to vary across religions, social groups, occupation of the household head, literacy, income and landholding pattern (National Institute of Nutrition, 2012). We take into account these factors while running the regressions.

We control for the household characteristics such as social groups (STs, SCs, Other Backward classes (OBC) and others), primary occupation of the household (self-employed in agriculture, self-employed in non-agriculture and others), religion (Hinduism, Islam, Christianity and others), size of the household, share of children in the age group of 0–6 and 7–14, land size class (dummy variables for 8 landsize class: less than 0.01 ha, 0.01–0.40 ha, 0.41–1.00 ha, 1.01–2.00 ha, 2.01–4.00 ha, 4.01–10.00, greater than 10 ha), MPCE decile classes, dummy variables for gender (male or female) and educational attainment of the household head (up to primary schooling or none, up to middle school; up to secondary school; above secondary school), sources of cooking (clean, dirty and others) and lighting (electricity or gas, kerosene and others) and whether there is a salaried member in the household.

## 5 Estimation results

Results from the DID regressions are presented in Table 6. Estimates as reported in column (1) were arrived at by controlling for the district fixed effects but not for the household characteristics. In the column (2), both district fixed effects and the household characteristics were controlled for. Since, the average treatment effects as reported by the DID estimate does not take into account the heterogeneity of the program effect, results from a quantile DID are also reported in order to understand the differential treatment effects across the distribution. Columns (3)–(5) report results from the quantile DID regression at the 25th, 50th and 75th quantile.^12^ The results suggest that the universal PDS in KBK region has led to an 8 percent (column 1) increase in per-capita intake of calories. Similarly, the per-capita protein and fat-intake increased by 8 and 10 percent respectively in the KBK districts. Coefficients on the time dummy is positive for nutrients (calorie, protein and fat), but the KBK region dummy is found be statistically insignificant.

One can see from the column (2) that on account of a universal PDS, there has been 7 percent increase in the consumption of calories and protein while fat intake has increased by 11 percent in the KBK region (column 1).^13^ Increase in the intake of calorie from non-cereals (20 percent) is larger than that of cereals (21 percent). Since, cereals are supplied through the PDS, we expect coefficient to be positive and significant which we do find.^14^ We also find a greater gain in the consumption of non-cereals which suggests a greater diversity in the diet. Looking at the coefficients in the case of various calorie sources as the outcome variables, we find a greater consumption of calorie from pulses, animal proteins, fruits and vegetables and edible oil. There has been 42 percent increase in the calorie from pulses in the diet, 27 percent in the case of calories from eggs, fish and meat, 33 percent from vegetables and fruits and 27 percent from the edible oils. No increase in the consumption of dairy products is found. The quantile DID estimates (columns (3–5) in Table 6) suggest a greater impact on the intake of calorie and fat for the lowest quartile. Increase in the consumption of protein however remains constant across the quartiles. There has been 44 percent increase in calories from pulses for those in the lowest quartile. This decreases monotonically as one goes up the higher MPCE classes. A similar patterns is observed for calorie from eggs, fish and meat, and vegetables and fruits.

### 5.1 Ratio of nutrient intake and the RDA

Summary statistics suggest that households in the KBK districts of Odisha fall well short of their recommended nutrient intake. To investigate whether a universal PDS in the KBK region furthered their progression towards their RDA of calories, protein and fat, we run separate DID regression with the percentage of RDA as the outcome variable which is calculated as the ratio of current nutrient intake to the RDA multiplied by hundred.^15^ Results are presented in Table 7. It suggests that the gap between the actual nutrient intake and the recommended one in the KBK region has come down by 4.94 percentage points for calories and 6.37 percentage points for protein. At the mean, we do not find any significant change for fat, though we do find significant increase in the consumption of fat for those in the lowest quartile.

### 5.2 Summary indices approach

Since we are testing for the significance of a large number of dependent variables, it might lead to higher probability of Type I errors leading to false rejection of the null hypothesis. To control for this bias, we use a summary indices approach as used by Clingingsmith et al. (2009) and (Kling et al., 2004).^16^ This summary index reduces combine together the multiple outcome measures into a single index which is a weighted mean of the standardized outcome variables. The weights are calculated such that the amount of information captured in the index is maximized by allowing for covariance across estimates through a seemingly-unrelated regression (SUR) framework. We group macronutrients (calorie, protein and fat) into one group and sources of calories into other group and estimate the DID regression as specified in Eq. (2). The results suggest that the macronutrient consumption increased by 32 percent for the KBK districts between 2004–05 and 2011–12 (Table 8). For the calorie sources, the index exhibits an increase of 37 percent. The ratio of nutrient intake to the RDA, suggest a 19 percent increase overall.

### 5.3 Triple DID estimates

Results from the triple DID approach as explained in Eqs. (3), (4) suggests that for the BPL households in the KBK region, universal PDS has not led to any significant increase in the consumption of calories, fat or protein as compared to the BPL households in the non-KBK region (Table 9). Though, we see that there is an increase in the consumption of calories from non-cereal food items, but the change is not significant for any specific non-cereal food group. It suggests that during the time when rapid expansion and improvements in PDS was taking place in Odisha and PDS in the KBK region was made universal, the nutritional intake of the BPL households was not found to be different across the KBK and non-KBK districts. Even when we look at the ratio of the nutrient intake to the RDA, there has been no significant difference between the BPL households in KBK district with respect to the non-KBK districts.

### 5.4 Robustness checks

To attribute this change in nutrient intake and dietary pattern in the KBK region to PDS, we test for the parallel trends assumption. Here, we use the 1999–2000 and 2004–05 data when there was no intervention in the KBK region. The absence of a statistically significant interaction term between time and the KBK dummy would suggest that there was no difference between the outcome variables for the KBK and non-KBK districts over time. Hence, the DID estimate would be unbiased and the increase between 2004–05 and 2011–12 could be attributed to the PDS. Results from these *placebo* regressions are reported in Table 10. We find that the common trend assumption holds for the calories and protein, but not for fat. Amongst sources of calories, it holds true only for the dairy products. For the DID regression where the ratio of nutrient intake to RDA is outcome variable, we find no change over time in the case of calorie protein and fat either at 1 percent or 5 percent of statistical significance. Here, we would like to mention again that there are issues of comparability over successive NSSO consumption expenditure surveys especially the 1999–00 round. Hence the comparison between 1999–00 and 2004–05 could potentially bias the results from our common trends assumption.^17^

One must be careful while interpreting the results from the parallel trends regressions in terms of any causal impact. If there has been a significant change in the outcome variables for the KBK districts over time, attributing this change to the PDS expansion would be misleading. In the present case, we find no change over the pre-intervention period for calorie and protein intake. This suggests that PDS did have an effect in increasing nutrient intake in the KBK districts of Odisha. Similarly, improvements in the ratio of nutrient intake to RDA can be attributed to PDS. Though, we cannot say the same for the different calorie sources as their consumption pattern do not follow the same trend.

#### 5.4.1 Households without ration cards as the control group

Despite having similar characteristics, all households in the KBK region do not have access to PDS as some of them do not possess a ration card. Since, these households are similar on most observable characteristics in the baseline period, we take the households without the ration card as an alternative control group.^18^ The sample is restricted to the KBK region only and we run a DID regression as specified in Eq. (2). The results we find are quite similar (Table 11). Calorie consumption of the households which had a PDS card in the KBK districts increased by 12 percent relative to the other households. Similar increase is evident in the case of protein and fat whose consumption went up by 10 and 20 percent respectively. In terms of calorie sources, there is a significant increase in the consumption of calorie from pulses (26 percent), edible oil (24 percent) and other products (34 percent). In the KBK districts, those with a PDS card were found to be 4.94 percent and 6.37 percent closer to their recommended calorie and protein intake.

#### 5.4.2 Non-poor households as the control group

To check for further robustness of our results, we run a DID regression with APL and no card households as the treated group and restrict our sample only to the KBK districts.^19^ We do so since the universalization of PDS in the KBK districts mostly benefitted the APL households. This is also clearly evident from Fig. 3. The results suggest that poorer households (BPL and AAY) benefitted more than the non-poor (APL and no ration card holders) households over time in terms of their nutrient intake and diet quality (Table 12). This could be explained by the fact that the poorer households too benefited from improved functioning of PDS post universalization. In fact, the poorer households benefitted more than the non-poor on account of this intervention.

#### 5.4.3 Propensity score matching with difference-in-difference regressions

Universal PDS scheme in KBK districts was implemented on account of high levels of poverty and food insecurity in the region. This makes it a non-random program placement where selection into treatment depends upon unobservable factors together with the observable factors. Such a non-random program placement could potentially bias the DID estimates, since the DID approach assumes that control and treatment groups are randomly assigned. To check for the robustness of our DID estimates, we combine propensity score matching (PSM) with the DID regressions (PSM-DID henceforth). PSM-DID has its advantages over the standard DID regressions as it controls for the bias arising out of the non-random program participation by controlling for the time-invariant unobserved characteristics affecting participation (Khandker et al., 2009). In the PSM-DID method, households in the pre-intervention period are ranked based upon their propensity scores and matched across the treatment and control groups. These propensity scores are calculated as the probability of the household being treated controlling for their observed characteristics. Matching of households is to identify the closest comparison group from the non-participant sample to the program participants. Hence, only those households above a certain propensity scores are considered for the analysis.

Here, households in the KBK districts are matched on all the observables and compared with similar households in the non-KBK districts. Similar approach has also been used by Kochar (2005) and Kaushal and Muchomba (2015) while estimating impact of PDS on nutrient intake. As a prelude to the PSM-DID regressions, we do a comparison of the outcome variables and a balancing *t-test* for the controls used in the regressions to see if the covariates on the pre-intervention period were statistically different across the treatment and control groups as suggested by Spears and Lamba (2013). We do not find any significant difference across the observed covariates while there is a significant difference between the nutrient intake across the KBK and non-KBK districts in the pre-intervention period 2004–05 (Table 13).

Upon matching households across the KBK and non-KBK districts on the basis of observables (caste, religion, household type, household size, asset index, total land possessed, dependency ratio, education and age of the household head, source of cooking/lighting and whether the household earns a regular salary income), we find a common support of 95 percent.^20^ Then, we estimate the DID specification as given by Eq. (2) for these households and the results are provided in Table 14. All the coefficients are positive and significant suggesting the positive impact of PDS on nutrient intake in the KBK districts over time as compared to the non-KBK districts (Table 14). These coefficients are slightly lower than the DID results in Table 6. Change in the consumption of calories from milk was not be found to be significant in Table 6, but here it is positive and significant, though at 90 percent level of confidence. Similar to the above specification, we run another PSM-DID regression by restricting our sample only to those households which consume from the PDS in the KBK districts only.^21^ Here again, we find there has been a significant increase in the nutrient intake over time for the households consuming rice from PDS compared to those who do not (Table 15). Also, we find a much stronger result in the case of households moving closer to their RDAs in the KBK region for calorie, protein and fat as well.

## 6 Concluding remarks

The role of nutritional support programs in the form of consumer food subsidy is considered an effective tool for offsetting the impact of hunger and poverty on nutrient intake and dietary quality. In this paper, we contribute to the literature on plausible impacts of food assistance programs like the PDS on nutrient intake and dietary quality. By comparing how outcomes have changed in two regions in Odisha- one with a targeted scheme and another with a universal PDS entitlement, we find a positive impact of PDS on nutrient intake. In the KBK region with a universal PDS, macronutrient consumption (calorie, protein and fat) increased by 32 percent between 2004–05 and 2011–12. The expansion of PDS coverage in this region also led to an improvement in the dietary quality which improved by 37 percent during the same period. Also, the ratio of macronutrient intake to the RDA increased by 19 percent in the KBK districts.

This brings us to the question whether these results could be generalized across other states of India and whether a universal PDS would work better. We would like to exercise caution here by saying that the chosen region of study here is amongst the poorest and most nutrient deficient households in the country. Having said that, our findings do have crucial policy implications for understanding nutrition based poverty traps and targeting of food subsidies. It also has implications for the National Food Security Act, 2013 under which the PDS is set to expand. Since, greater income only may not lead to improved nutrition as households may substitute away from more nutritive food items to tasty but less nutritive food items. An enabling food environment in the form of consumer food subsidy goes a long away in ensuring the consumption of a minimum amount of calories when food price fluctuations are high. In Odisha, especially the KBK districts of the state with high levels of poverty and malnutrition, PDS has played a crucial role in reducing hunger with greater availability of foodgrains at extremely low prices. This has been brought about by making PDS entitlements universal which not only lowers the incentive for leakages in the system, but also fosters greater political support for the scheme. It has been argued in the literature on political economy of social transfers that targeted schemes work better when benefits are also bestowed upon the non-poor households. In this case as well, there has been an overall improvement in nutrient intake when PDS entitlements were provided to the non-poor households.

We make no claim that these results could be generalized and the expansion of PDS coverage to 75 percent of the rural and 50 percent of the rural population under the National Food Security Act, 2013 would improve nutrient intake. On the issue of a targeted versus universal PDS, the only claim we make is the expanded coverage to the APL households in KBK districts benefitted BPL households as well which corroborates the existing evidence from other states like Tamil Nadu and Himachal Pradesh where expanded PDS coverage has benefitted all, especially the poor.

## Figures and Tables

**Table A1:** ICMR recommended RDA by age and gender.

	Age group	Calorie	Fat	Protein
Man		2320	60	25
Woman		1900	55	20
Infants	<1 year	92	1.16	0
Children	1–3 years	1060	16.7	27
Children	4–6 years	1350	20.1	25
Children	7–9 years	1690	29.5	30
Boys	10–12 years	2190	39.9	35
Girls	10–12 years	2010	40.4	35
Boys	13–15 years	2750	54.3	45
Girls	13–15 years	2330	51.9	40
Boys	16–17 years	3020	61.5	50
Girls	16–17 years	2440	55.5	35

**Table A2:** Adult equivalent conversion factors.

Age (in years)	<1	1–3	4–6	7–9	10–12	13–15	16–19	20–39	40–49	50–59	60–69	>70
Male	0.43	0.54	0.72	0.87	1.03	0.97	1.02	1	0.95	0.9	0.8	0.7
Female	0.43	0.54	0.72	0.87	0.93	0.8	0.75	0.71	0.68	0.64	0.51	0.5

**Table A3:** Existing studies on impact of PDS on nutrient intake.

Paper	Data used	Assumptions	Empirical methods	Conclusion
Kochar (2005)	1993–94 and 1999–00 17 major states	In the absence of information of BPL cards, they use the predicted probability of owning one based upon observables	OLS, IV-2SLS (predicted probability for BPL cards for BPL households)	PDS subsidy had no impact on the intake of calories

Kaushal and Muchomba (2015)	1993–94; 1999–00; 2004–05 All major states	In the absence of information of BPL cards, they use the predicted probability of owning one based upon observables	OLS, IV-2SLS (predicted probability for BPL cards for BPL households)	PDS subsidy has negligible impact on calorie intake; Modest impact on consumption patterns

Kishore and Chakrabarti (2015)	2004–05; 2009–10 Andhra Pradesh, Chhattisgarh, Odisha, Tamil Nadu and West Bengal	In the absence of information of BPL cards, they consider the bottom 20% of households as poor	Difference-in-Difference (DID); Triple DID	Savings from the subsidized rice were used to spend on pulses, edible oil, vegetables and sugar

Kaul (2014)	2002–2008 thin rounds Andhra Pradesh, Assam, Karnataka, Kerala, Orissa, Jharkhand, Chhattisgarh and West Bengal	No impact of PDS subsidy on calorie intake for those not consuming from the PDS as a counterfactual	OLS	PDS subsidy positively affects cereal consumption and overall calorie intake

Krishnamurthy et al. (2014a)	1999–00; 2004–05 Chhattisgarh	Consumption data from the 1999–00 round based upon the 7-day recall period is comparable with later rounds where consumption calculated based upon 30-day recall	Difference-in-Difference (DID)	PDS expansion led to greater nutrient intake and dietary quality in Chhattisgarh relative to other neighboring districts

This paper	2004–05; 2011–12 Odisha	Parallel trends assumptions hold for nutrient intake	Difference-in-Difference (DID); Triple DID	Expanded PDS coverage led to higher calorie intake and improvements in diet quality

^a^ All these papers use the NSSO data.

**Fig. A1:**
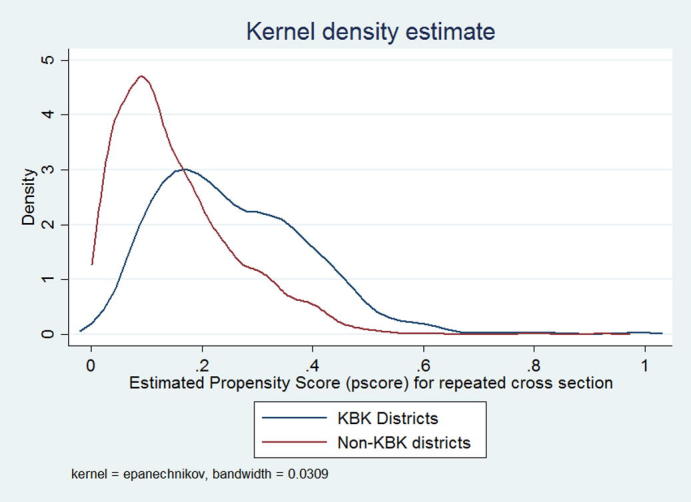
Common support for KBK and non-KBK households based upon the observables.

**Fig. A2:**
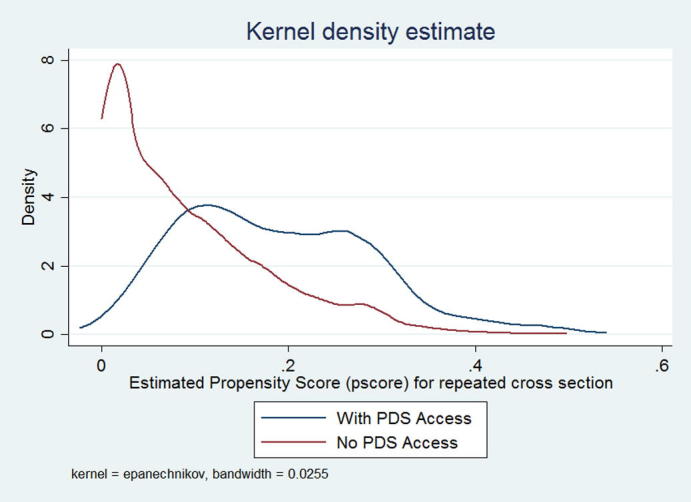
Area of common support for PSM (Consumption from PDS in the KBK districts).

**Table 1:** Household profile by types of ration card possessed (in %).

	Odisha		KBK Districts		Non-KBK Districts	
	2004–05	2011–12	2004–05	2011–12	2004–05	2011–12
AAY	1.99	5.49	2.71	6.24	1.85	5.36
BPL	42.57	47.86	48.94	58.08	41.29	46.04
APL	22.41	18.22	7.41	8.61	25.42	19.93
No card	33.02	28.43	40.95	27.07	31.43	28.67

AAY: Antayodaya Anna Yojana; BPL: Below Poverty Line; APL: Above Poverty Line.

Note: Sample frequency weights have been used to arrive at these estimates.

**Table 2:** Monthly rice consumption from PDS.

	KBK		Non-KBK	
	2004–05	2011–12	2004–05	2011–12
	*Monthly rice consumption from PDS (kg per household)*			
AAY	24.8	31.9	26.4	32.5
BPL	14.1	27.6	6.5	27.9
APL	6.4	21.9	0.6	2.2
No card	2.1	0.2	0.1	0.3

Total	8.9	20	3.3	15.1

	*Share of monthly rice consumption from PDS to total (in %)*			
AAY	47.6	58.8	45.2	55.5
BPL	30.9	59.8	9.6	50.1
APL	11.6	40.5	0.9	4.2
No card	4.5	0.45	0.2	0.8

Total	19	44.5	5.2	29.2

Note: Sample frequency weights have been used to arrive at these estimates.

**Table 3:** Average nutrient intake (per person per day).

	AAY		BPL		APL		No card		Total	
	2004–05	2011–12	2004–05	2011–12	2004–05	2011–12	2004–05	2011–12	2004–05	2011–12
*Non-KBK Districts*										
Calories (in kcal)	1945.0	2060.2	2013.6	2016.6	2246.1	2097.3	2014.0	2055.9	2076.8	2046.5
Fat (in g)	44.5	47.7	46.8	47.2	53.9	50.7	47.9	49.6	49.0	48.6
Protein (in g)	13.8	20.8	14.8	20.8	22.9	27.1	19.2	25.6	18.3	23.4
Non-cereal calories	319.7	407.8	356.1	429.9	536.2	543.8	456.9	526.0	433.6	477.6

*KBK Districts*										
Calories	1698.7	1800.0	1558.2	1819.9	1891.7	1950.5	1775.9	1768.9	1674.6	1819.0
Fat	37.3	42.0	36.0	42.0	44.8	46.7	41.0	41.7	38.7	42.4
Protein	9.1	14.8	10.2	17.4	16.2	22.2	15.5	20.2	12.7	18.4
Non-cereal calories	183.4	330.5	215.2	361.9	342.9	449.5	352.2	422.9	278.7	383.2

Note:

1. Calorie is in kilocalories. Proteins and fats are measured in grams.

2. Sample frequency weights have been used to arrive at these estimates.

**Table 4:** Difference in the mean nutrient consumption over 2004–05 and 2011–12.

	Odisha			KBK Districts			Non-KBK Districts		
	All	BPL	Non-BPL	All	BPL	Non-BPL	All	BPL	Non-BPL
*Major macronutrients*									
Calories	11.1	77.3⁎⁎⁎	−24.655	132.3⁎⁎⁎	237.8⁎⁎⁎	63.7	−11.9	45.1⁎⁎	−42.9⁎⁎
Protein	0.7⁎	2.0⁎⁎⁎	0.252	3.7⁎⁎⁎	5.8⁎⁎⁎	2.7⁎⁎	0.1	1.1⁎⁎	−0.2
Fat	5.8⁎⁎⁎	6.66⁎⁎⁎	5.929⁎⁎⁎	5.3⁎⁎⁎	7.2⁎⁎⁎	4.8⁎⁎⁎	5.9⁎⁎⁎	6.6⁎⁎⁎	6.0⁎⁎⁎

*Sources of calories*									
Cereals	−40.9⁎⁎⁎	−17.6	62.9⁎⁎⁎	75.2⁎⁎⁎	91.9⁎⁎⁎	54.6	63.6⁎⁎⁎	−40.2⁎⁎	−85.0⁎⁎⁎
Non-cereals	52.0⁎⁎⁎	94.9⁎⁎⁎	38.2⁎⁎⁎	57.1⁎⁎	145.8⁎⁎⁎	9.1	51.7⁎⁎⁎	85.3⁎⁎⁎	42.1⁎⁎⁎
Pulses	17.6⁎⁎⁎	22.6⁎⁎⁎	15.8⁎⁎⁎	32.2⁎⁎⁎	37.4⁎⁎⁎	30.2⁎⁎⁎	14.6⁎⁎⁎	19.1⁎⁎⁎	13.1⁎⁎⁎
Egg, fish & meat	1.0⁎⁎	1.7⁎⁎⁎	0.9	4.1⁎⁎⁎	4.6⁎⁎⁎	4.2⁎⁎⁎	0.4	1.1⁎	0.2
Dairy products	19.6⁎⁎⁎	18.0⁎⁎⁎	24.6⁎⁎⁎	13.7⁎⁎⁎	12.5⁎⁎⁎	22.4⁎⁎⁎	20.9⁎⁎⁎	19.7⁎⁎⁎	24.8⁎⁎⁎
Vegetables and fruits	−15.8⁎⁎⁎	11.9⁎⁎⁎	−16.6⁎⁎⁎	10.1⁎⁎⁎	10.2⁎⁎⁎	14.0⁎⁎⁎	20.8⁎⁎⁎	−16.0⁎⁎⁎	22.5⁎⁎⁎
Edible oil	44.7⁎⁎⁎	47.6⁎⁎⁎	45.8⁎⁎⁎	47.9⁎⁎⁎	54.3⁎⁎⁎	46.1⁎⁎⁎	44.2⁎⁎⁎	46.4⁎⁎⁎	45.3⁎⁎⁎
Other food items	−15.0⁎⁎	16.8⁎⁎⁎	−32.3⁎⁎⁎	−51.1⁎⁎	26.8⁎⁎	−108.0⁎⁎	−7.6	14.8⁎⁎⁎	−18.8⁎

Note:

1. BPL includes AAY households as well.

2. Sample frequency weights have been used to arrive at these estimates.

⁎⁎⁎ Significance at 1 percent.

⁎⁎ Significance at 5 percent.

⁎ Significance at 10 percent.

**Table 5:** Percentage of households below their Recommended Dietary Allowance (RDA) by MPCE quartiles.

MPCE Deciles	Calories				Protein				Fat			
	KBK Districts		Non-KBK Districts		KBK Districts		Non-KBK Districts		KBK Districts		Non-KBK Districts	
	2004–05	2011–12	2004–05	2011–12	2004–05	2011–12	2004–05	2011–12	2004–05	2011–12	2004–05	2011–12
Bottom	100	99.42	95.89	95.98	99.08	95.76	87.43	89.34	100	100	99.93	99.22
25–50	99.69	94.95	80	83.89	97.26	81.2	67.45	66.88	100	100	98.82	89.62
50–75	85.55	81.62	63.95	75.82	78.67	73.73	43.53	52.74	97.65	92.63	95.88	72.19
Top	74.7	80.31	38.63	55	61.92	67.45	17.01	31.31	80.91	69.56	57.57	39.16
Total	89.93	89.01	69.61	77.66	84.16	79.49	53.84	60.05	94.6	90.5	88.05	75.03

Note: Sample frequency weights have been used to arrive at these estimates.

**Table 6:** Difference in difference estimates.

	Without covariates and District Fes	With Covariates and District FEs			
		Quantiles		
	OLS	OLS	0.25	0.5	0.75
	(1)	(2)	(3)	(4)	(5)
*Macronutrients*					
Calorie	0.08⁎⁎⁎	0.07⁎⁎⁎	0.09⁎⁎⁎	0.06⁎⁎⁎	0.06⁎⁎⁎
	(0.02)	(0.02)	(0.02)	(0.01)	(0.01)
Protein	0.08⁎⁎⁎	0.07⁎⁎⁎	0.07⁎⁎⁎	0.07⁎⁎⁎	0.07⁎⁎⁎
	(0.02)	(0.02)	(0.02)	(0.01)	(0.01)
Fat	0.10⁎⁎⁎	0.11⁎⁎⁎	0.14⁎⁎⁎	0.09⁎⁎⁎	0.10⁎⁎⁎
	(0.03)	(0.02)	(0.02)	(0.03)	(0.03)

*Sources of calories*					
Cereals	0.21⁎⁎⁎	0.17⁎⁎⁎	0.11⁎⁎⁎	0.05⁎⁎⁎	0.05⁎⁎⁎
	(0.04)	(0.04)	(0.02)	(0.02)	(0.02)
Non-cereals	0.18⁎⁎⁎	0.20⁎⁎⁎	0.23⁎⁎⁎	0.21⁎⁎⁎	0.21⁎⁎⁎
	(0.03)	(0.02)	(0.03)	(0.02)	(0.02)
Pulses	0.47⁎⁎⁎	0.42⁎⁎⁎	0.44⁎⁎⁎	0.34⁎⁎⁎	0.33⁎⁎⁎
	(0.06)	(0.05)	(0.06)	(0.05)	(0.04)
Milk	−0.07	0.04	−0.08	−0.14⁎	0.22
	(0.13)	(0.10)	(0.06)	(0.08)	(0.15)
Eggs, fish and meat	0.30⁎⁎⁎	0.27⁎⁎⁎	0.47⁎⁎⁎	0.26⁎⁎⁎	0.17⁎⁎⁎
	(0.07)	(0.06)	(0.11)	(0.07)	(0.06)
Vegetables & fruits	0.34⁎⁎⁎	0.33⁎⁎⁎	0.31⁎⁎⁎	0.30⁎⁎⁎	0.21⁎⁎⁎
	(0.04)	(0.03)	(0.03)	(0.03)	(0.03)
Edible oil	0.28⁎⁎⁎	0.27⁎⁎⁎	0.19⁎⁎⁎	0.17⁎⁎⁎	0.20⁎⁎⁎
	(0.04)	(0.04)	(0.04)	(0.04)	(0.03)
Others	0.10⁎⁎	0.11⁎⁎⁎	0.17⁎⁎⁎	0.11⁎⁎⁎	0.16⁎⁎⁎
	(0.05)	(0.04)	(0.04)	(0.04)	(0.05)

Notes:

1. The covariates used in the estimates for columns (2)–(4) are the household social groups (ST, SC, OBC and others), household type, religion, size of the household, percentage of children in the age group of 0–6 and 7–14, land size class, gender and educational attainment of the household head, sources of cooking and lighting and whether the household has a salaried member. Standard errors are provided in parentheses.

2. Robust standard errors for the quantile DID estimates have been arrived at by bootstrapping them 50 times.

⁎⁎⁎ p < 0.01.

⁎⁎ p < 0.05.

⁎ p < 0.1.

**Table 7:** DID regression: ratio of nutrient intake to RDA.

		Quantile estimates		
	OLS	0.25	0.5	0.75
	(1)	(2)	(3)	(4)
Calories	4.94⁎⁎⁎	6.55⁎⁎⁎	5.11⁎⁎⁎	5.72⁎⁎⁎
	(1.22)	(1.31)	(1.15)	(1.4)

Protein	6.37⁎⁎⁎	4.61⁎⁎⁎	6.13⁎⁎⁎	8.68⁎⁎⁎
	(1.49)	(1.24)	(1.15)	(1.41)

Fat	1.43	2.92⁎⁎	1.71	2.37
	(2.58)	(1.31)	(1.50)	(2.03)

Notes:

1. The outcome variable in the nutrient intake per adult equivalent in the household divided by the RDA for each household. This ratio is multiplied by 100 for the results to be interpreted in percentage terms. 2. The covariates used in the estimation are the household social groups (ST, SC, OBC and others), household type, religion, size of the household, percentage of children in the age group of 0–6 and 7–14, land size class, gender and educational attainment of the household head, sources of cooking and lighting and whether the household has a salaried member.

2. Robust standard errors for the quantile DID estimates have been arrived at by bootstrapping them 50 times.

⁎⁎⁎ p < 0.01.

⁎⁎ p < 0.05. ^⁎^ p < 0.1.

**Table 8:** DID estimates from the summary index approach.

	Average effect	Std. error.
Macronutrients	0.32⁎⁎⁎	0.03
Source of calorie	0.37⁎⁎⁎	0.03
Ratio of macronutrient intake to RDA	0.19⁎⁎⁎	0.03

Note:

1. Macronutrients comprise an index of calorie, protein and fat.

2. Sources of calories include consumption of cereals, pulses, eggs fish and meat, milk, edible oil, vegetable and fruits and other items.

3. The explanatory variables are the same as in other regression.

⁎⁎⁎ p < 0.01. ^⁎⁎^ p < 0.05. ^⁎^ p < 0.1.

**Table 9:** Triple DID estimates.

	DID	Std. errors
*Macronutrients*		
Calorie	0.03	(0.03)
Protein	0.02	(0.03)
Fat	0.07	(0.05)

*Sources of calories*		
Cereal	−0.09	(0.08)
Non-cereal	0.12⁎⁎⁎	(0.04)
Pulses	0.12	(0.11)
Milk	−0.09	(0.21)
Eggs, fish and meat	−0.12	(0.13)
Vegetables and fruits	−0.11	(0.07)
Edible oil	0.08	(0.08)
Others	0.11	(0.07)

*Ratio of nutrient intake to RDA*		
Calorie	3.12	(2.43)
Protein	2.29	(2.99)
Fat	2.40	(5.17)

1. The triple DID coefficient, $\tau_{\mathrm{DID}}^{\mathrm{tr}}$ is for the interaction term, $T_{d}*t_{i}*{\mathrm{BPL}}_{i}$ as presented in Eqs. (3), (4).

2. The covariates used in the estimation are the household social groups (ST, SC, OBC and others), household type (self-employed in agriculture, self-employed in non-agriculture and others), religion, size of the household, percentage of children in the age group of 0–6 and 7–14, land size class, gender and educational attainment of the household head, sources of cooking and lighting and whether the household has a salaried member. Standard errors are provided in parentheses. The estimates are arrived at controlling for the district fixed effects.

3. The dependent variables are the natural logarithmic transformation of the per-capita daily values.

⁎⁎⁎ p < 0.01. ^⁎⁎^ p < 0.05. ^⁎^ p < 0.1.

**Table 10:** Results from the placebo DID.

	Coeff	Std. errors
*Macronutrients*		
Calories	0.01	(0.02)
Protein	−0.01	(0.02)
Fat	−0.10⁎⁎⁎	(0.03)
Non-cereals	−0.14⁎⁎⁎	(0.03)

*Source of calories*		
Cereals	−0.16⁎⁎⁎	(0.05)
Pulses	−0.37⁎⁎⁎	(0.08)
Milk	0.14	(0.12)
Eggs, fish and meat	−0.40⁎⁎⁎	(0.08)
Edible oil	−0.36⁎⁎⁎	(0.05)
Vegetables & fruits	−0.52⁎⁎⁎	(0.04)
Others	0.14⁎⁎⁎	(0.05)

*Ratio of nutrient intake to RDA*		
Calories	−2.72	(1.72)
Protein	−4.47⁎	(2.68)
Fat	−7.29	(5.63)

Notes:

1. The coefficients reported here are from the interaction terms between the earlier period 1999–00 and 2004–05 and the KBK region dummy during which no intervention took place.

2. Covariates used in the estimation are the household social groups (ST, SC, OBC and others), household type, religion, size of the household, percentage of children in the age group of 0–6 and 7–14, land size class, gender and educational attainment of the household head, sources of cooking and lighting and whether the household has a salaried member.

3. Standard errors are provided in parentheses.

⁎⁎⁎ p < 0.01. ^⁎⁎^ p < 0.05.

⁎ p < 0.1.

**Table 11:** DID estimates with no ration card in the KBK region as the control group.

	DID	Std. errors
*Macronutrients*		
Calorie	0.12⁎⁎⁎	(0.03)
Protein	0.10⁎⁎⁎	(0.03)
Fat	0.20⁎⁎⁎	(0.04)

*Sources of calories*		
Cereal	0.07	(0.07)
Non-cereal	0.26⁎⁎⁎	(0.04)
Pulses	0.44⁎⁎⁎	(0.11)
Milk	0.27	(0.18)
Eggs, fish and meat	0.15	(0.12)
Vegetables and fruits	0.02	(0.05)
Edible oil	0.24⁎⁎⁎	(0.08)
Others	0.37⁎⁎⁎	(0.08)

*Ratio of nutrient intake and RDA*		
Calorie	9.07⁎⁎⁎	(2.14)
Protein	8.79⁎⁎⁎	(2.61)
Fat	7.43⁎⁎	(3.14)

Notes:

1. The results are only for the KBK sample. Treatment group constitutes households with any ration card (AAY/BPL/APL) while the treatment group comprises those households who do not have a ration card.

2. The covariates used in the estimation are the household social groups (ST, SC, OBC and others), household type (self-employed in agriculture, self-employed in non-agriculture and others), religion, size of the household, percentage of children in the age group of 0–6 and 7–14, land size class, gender and educational attainment of the household head, sources of cooking and lighting and whether the household has a salaried member. Standard errors are provided in parentheses.

3. The dependent variables are the natural logarithmic transformation of the per-capita daily values.

⁎⁎⁎ p < 0.01.

⁎⁎ p < 0.05. ^⁎^ p < 0.1.

**Table 12:** DID estimates for the KBK sample with non-BPL card holders as the treatment group.

	Coefficients	Std. errors
*Macronutrients*		
Calorie	−0.10⁎⁎⁎	(0.03)
Protein	−0.09⁎⁎⁎	(0.03)
Fat	−0.22⁎⁎⁎	(0.04)

*Sources of calories*		
Cereal	−0.04	(0.07)
Non-cereal	−0.28⁎⁎⁎	(0.04)
Pulses	−0.48⁎⁎⁎	(0.10)
Milk	−0.29	(0.18)
Eggs, fish and meat	−0.24⁎⁎	(0.12)
Vegetables and fruits	−0.05	(0.05)
Edible oil	−0.30⁎⁎⁎	(0.08)
Others	−0.36⁎⁎⁎	(0.07)

*Ratio nutrient intake and RDA*		
Calorie	−6.43⁎⁎⁎	(2.10)
Protein	−6.12⁎⁎	(2.56)
Fat	−7.52⁎⁎	(3.05)

Notes:

1. The results are only for the KBK sample. Treatment group constitutes non-BPL households (APL/No card holders) while the treatment group comprises those households who possess either a AAY/BPL card.

2. The covariates used in the estimation are the household social groups (ST, SC, OBC and others), household type (self-employed in agriculture, self-employed in non-agriculture and others), religion, size of the household, percentage of children in the age group of 0–6 and 7–14, land size class, gender and educational attainment of the household head, sources of cooking and lighting and whether the household has a salaried member. Standard errors are provided in parentheses.

3. The dependent variables are the natural logarithmic transformation of the per-capita daily values.

⁎⁎⁎ p < 0.01.

⁎⁎ p < 0.05. ^⁎^ p < 0.1.

**Table 13:** Comparison of the outcome variables and the covariates in the pre-intervention period, 2004–05.

	Non-KBK	KBK	Difference	t-stats	Pr(T > t)
*Outcome variables*					
Calories	2159.3	1801.4	−357.9	13.7	0.0⁎⁎⁎
Protein	51.4	41.7	−9.7	13.7	0.0⁎⁎⁎
Fat	21.3	15.7	−5.5	8.3	0.0⁎⁎⁎

*Sources of calorie*					
Cereals	1649.4	1416.9	−232.6	11.6	0.0⁎⁎⁎
Non-cereals	509.8	384.5	−125.3	7.2	0.0⁎⁎⁎
Pulses	66.5	46.3	−20.2	10.1	0.0⁎⁎⁎
Egg, fish & meat	17.7	9.6	−8.1	8.3	0.0⁎⁎⁎
Dairy products	40.3	25.3	−15.0	4.8	0.0⁎⁎⁎
Vegetables and fruits	145.0	70.5	−74.5	21.5	0.0⁎⁎⁎
Edible oil	101.4	70.1	−31.3	12.4	0.0⁎⁎⁎
Other food items	139.0	162.8	23.9	1.7	0.0⁎

*Control variables*					
*Social group* [STs = 0]					
SCs	0.2	0.2	0.0	0.9	0.3655
OBCs	0.3	0.3	0.0	0.1	0.9176
Others	0.1	0.1	0.0	0.6	0.5214

*Religion [Hinduism* = *0]*					
Islam	0.0	0.0	0.0	0.9	0.3722
Christianity	0.0	0.0	0.0	0.5	0.5862
*Household size*	4.4	4.3	−0.1	1.6	0.1181

*Household type [Self-Employed in Agriculture *=* 0]*					
Self-employed in non-agriculture	0.2	0.2	0.0	1.0	0.3002
Other	0.5	0.5	0.0	1.3	0.1807
Asset index	−1.0	−1.0	0.0	0.3	0.7384

*Land size class [Base category *=* 0–0.01 ha]*					
0.01–0.40 ha	0.2	0.2	0.0	0.1	0.9418
0.41–1.00 ha	0.2	0.2	0.0	1.1	0.2954
1.01–2.00 ha	0.2	0.2	0.0	1.0	0.3003
2.01–4.00 ha	0.1	0.1	0.0	1.1	0.2691
4.01–10.00 ha	0.1	0.1	0.0	0.5	0.6142
>10 ha	0.0	0.0	0.0	0.8	0.3986
Share of children < 6 yrs	14.1	14.9	0.8	1.0	0.3074
Share of children 7–14 yrs	14.7	14.3	−0.4	0.7	0.4967

*Gender of the household head [Male* = *0]*					
Female	0.1	0.1	0.0	0.0	0.9656

*Education of the household head [ >Primary/no education* = *0]*					
Upto primary	0.0	0.0	0.0	0.0	0.9689
Upto middle	0.0	0.0	0.0	0.3	0.7406
Upto secondary	0.0	0.0	0.0	0.2	0.8397
Above secondary	0.0	0.0	0.0	0.2	0.8507
Age of the household head	42.3	42.5	0.1	0.3	0.7968
*Cooking source* [Clean = 0]					
Dirty	0.9	0.9	0.0	0.6	0.5463
Others	0.0	0.0	0.0	0.6	0.5294

*Lighting source [Electricity/Gas *=* 0]*					
Kerosene and others	0.7	0.7	0.0	0.2	0.8536
*Regular salaried* [Yes = 0]					
No	0.9	0.9	0.0	0.1	0.9008

*MPCE Decile Class [0–10 Decile Class *=* 0]*					
10–20	0.1	0.1	0.0	0.6	0.5266
20–30	0.1	0.1	0.0	0.0	0.9662
30–40	0.1	0.1	0.0	0.4	0.7284
40–50	0.1	0.1	0.0	0.5	0.642
50–60	0.1	0.1	0.0	0.8	0.4541
60–70	0.1	0.1	0.0	0.2	0.8734
70–80	0.1	0.1	0.0	0.7	0.476
80–90	0.1	0.1	0.0	0.5	0.5905
90–100	0.1	0.1	0.0	0.2	0.8454

Notes: 1. Calculations based upon the pre-intervention 2004–05 data.

⁎⁎⁎ p < 0.01. ^⁎⁎^ p < 0.05.

⁎ p < 0.1.

**Table 14:** PSM-DID estimates for matched households across KBK and non-KBK districts.

	PSM-DID estimates	Std. errors
*Macronutrients*		
Calorie	0.04⁎⁎⁎	0.02
Protein	0.05⁎⁎⁎	0.01
Fat	0.07⁎⁎⁎	0.02

*Sources of calories*		
Cereal	0.12⁎⁎⁎	0.04
Non-cereal	0.15⁎⁎⁎	0.02
Pulses	0.32⁎⁎⁎	0.06
Milk	0.15⁎	0.08
Eggs, fish and meat	0.2⁎⁎⁎	0.08
Vegetables and fruits	0.25⁎⁎⁎	0.03
Edible oil	0.19⁎⁎⁎	0.03
Others	0.09⁎⁎⁎	0.03

*Ratio nutrient intake and RDA*		
Calorie	2.55⁎	1.31
Protein	3.75⁎⁎	1.65
Fat	−0.1	0.00

1. Treatment group constitutes the KBK districts, while the non-KBK districts are the control group.

2. The PSM-DID estimates have been arrived at by matching households based upon caste, religion, household type, household size, asset index, total land possessed, dependency ratio, education and age of the household head, source of cooking/lighting and whether the household earns a regular salary income.

3. The dependent variables are the natural logarithmic transformation of the per-capita daily values.

⁎⁎⁎ p < 0.01.

⁎⁎ p < 0.05.

⁎ p < 0.1.

**Table 15:** PSM-DID estimates for households consuming rice from PDS within in the KBK districts.

	PSM-DID estimates	Std. errors
*Macronutrients*		
Calorie	0.07⁎⁎⁎	0.02
Protein	0.07⁎⁎⁎	0.02
Fat	0.16⁎⁎⁎	0.03

*Sources of calories*		
Cereal	0.02	0.02
Non-cereal	0.26⁎⁎⁎	0.03
Pulses	0.39⁎⁎⁎	0.08
Milk	0.18⁎⁎	0.08
Eggs, fish and meat	0.18⁎⁎	0.07
Vegetables and fruits	0.16⁎⁎⁎	0.04
Edible oil	0.24⁎⁎⁎	0.05
Others	0.21⁎⁎⁎	0.06

*Ratio nutrient intake and RDA*		
Calorie	5.3⁎⁎⁎	1.40
Protein	5.98⁎⁎⁎	1.68
Fat	3.13⁎⁎	1.38

This is restricted sample only to the KBK region.

Treatment group constitutes of the households which consume rice from PDS while rest of the households in the KBK districts act as the control group.

The PSM-DID estimates have been arrived at by matching households based upon caste, religion, household type, household size, asset index, total land possessed, dependency ratio, education and age of the household head, source of cooking/lighting and whether the household earns a regular salary income.

The dependent variables are the natural logarithmic transformation of the per-capita daily values.

⁎⁎⁎ p < 0.01.

⁎⁎ p < 0.05. ^⁎^ p < 0.1.

## Appendix A

Recommended Dietary Intake (RDA) of nutrients intake as published by the ICMR (2010) is converted into household level RDA which is further arrived at in terms of the consumer unit/adult equivalent level. The ICMR RDA is based upon the gender, nature of job and weight. Unfortunately, the NSS data does not collect any information on individual level nature of work and weight. Hence, we have taken an average of the dietary requirements. The RDA for different category of individuals is as follow (see Table A1):

To arrive at the individual level RDA, we divided the household RDA by the adult equivalent according to the following reports from the NSSO Reports (see Table A2, Table A3).

## Footnotes

1 Odisha has been one of the most proactive states in terms of restructuring the PDS and improving its effectiveness (Aggarwal, 2011).

2 Chatterjee (2014) provides qualitative evidence on how PDS has reduced the levels of experiential food insecurity in Koraput, one of the KBK districts.

3 We estimate rural poverty rate in southern Odisha stands highest at 48 percent followed by the northern (40 percent) and coastal regions (21 percent) in 2011–12. Districts along the eastern ghats with a higher share of tribal population are most food insecure (WFP and IHD, 2008).

4 KBK region comprised of 8 districts of Odisha namely – Malkangiri, Koraput, Nabrangpur, Kalahandi, Rayagada, Nuapada, Balangir and Sonepur. They constitute 30.59 percent of the total geographical area of the state. Close to 70 percent of the households either belong to the marginalized social groups (SCs/STs) in some of these districts. The primary occupation in these districts is agriculture, with a majority of the families working as agricultural labor (Census of India, 2011). Lack of inadequate infrastructure such as road connectivity also acts as a major constraint. Declining forest cover on account of intensive use, shifting cultivation and mining has adversely impacted the traditional sources of livelihood.

5 There were two survey schedules used in 2011–12 based upon the 30 days and 7 days recall period. This article uses the 30 days schedule for the sake of comparability over time.

6 There was a slight change in the survey questionnaire over time. The 2004–05 survey did not take into account the consumption of free meals. Since free meals constitute a very small part of the daily intake, nutrient intake from free meals has been removed for the sake of comparability here.

7 RDA varies by gender, weight and nature of work of an individual. However, in this dataset, we do not observe individual characteristics like their weight and the nature of work. In the appendix to this paper, we have described how household level RDA is calculated within the constraints of data availability. Weights for the consumer units according to their age profile are also provided in the appendix.

8 Non-possession of ration cards could be a result of targeting errors or households opting out of the scheme if they consider themselves non-poor. In our sample, 39.4 percent of the households who do not possess a ration card can be classified as poor as per the official poverty line of Rs. 407.78 monthly per-capita expenditure at the 2004–05 prices.

9 The share of rice from PDS in rural Odisha has increased from 7% to 31% during 2004–05 and 2011–12 (Rahman, 2014).

10 Unit values here refers to the number arrived at by dividing the total expenditure to the quantity purchased by each household.

11 Deaton and Drèze (2009) have argued that the decline in the energy intake is more or in account of changing nature of occupation which require less manual labor. An improvement in the disease environment and the quality of sanitation requirement has further lowered nutrient requirements (Duh and Spears, 2015).

12 It has been widely recognized that the treatment effects of an intervention are not homogeneous (Ravallion, 2007).

13 One of the referees suggested we estimate a similar DID regression for the whole of Odisha with BPL+AAY households as the treatment group and the nonpoor households as the control group. The results suggest that nutrient intake for the poorer households relative to those above the poverty line has increased significantly between 2004–05 and 2011–12.

14 Of course, we can have a negative coefficient when the outcome variable is calorie from cereals if the total consumption of rice and wheat declines.

15 For example, if the per adult equivalent RDA for calories is 100 kcal and the individual calorie intake is 80 kcal, it implies that the individual consumes 80% of the RDA.

16 We thank one of the referees for pointing this out and suggesting a reference to the relevant literature.

17 Infact, the data shows a decline in the calorie intake between 1999–00 and 2004–05 in Table 10, which suggests an overestimation of consumption in the 1999–00 round. Surveys based upon the 7 day recall generally report higher levels of consumption as compared to the 30 days.

18 MPCE for households without any ration card is comparable to those of the APL households in the KBK region. Average MPCE for those with no ration card is Rs. 427 against Rs. 429 for the APL card holders in the KBK region. MPCE of the BPL households is much lower. We also did a *t*-test for all the observed covariates and did not find any significant difference between the treatment and control groups.

19 We are thankful to one of the referees for pointing this out.

20 Kernel density estimates for the common support are provided in the appendix (Fig. A1, Fig. A2).

21 Here again, we again we get quite high area of common support at 85% and the kernel density curves are provided in the appendix.
